# Isolation and Functional Characterization of a Salt-Responsive Calmodulin-Like Gene *MpCML40* from Semi-Mangrove *Millettia pinnata*

**DOI:** 10.3390/ijms22073475

**Published:** 2021-03-27

**Authors:** Yi Zhang, Jianzi Huang, Qiongzhao Hou, Yujuan Liu, Jun Wang, Shulin Deng

**Affiliations:** 1 Key Laboratory of South China Agricultural Plant Molecular Analysis and Genetic Improvement & Guangdong Provincial Key Laboratory of Applied Botany, South China Botanical Garden, Chinese Academy of Sciences, Guangzhou 510650, China; yizhang@scbg.ac.cn; 2 Guangdong Provincial Key Laboratory for Plant Epigenetics, College of Life Sciences and Oceanography, Shenzhen University, Shenzhen 518060, China; biohjz@szu.edu.cn; 3 College of Life Sciences, University of Chinese Academy of Sciences, Beijing 100049, China; houqiongzhao@scbg.ac.cn (Q.H.); liuyujuan@scbg.ac.cn (Y.L.); 4 CAS Engineering Laboratory for Vegetation Ecosystem Restoration on Islands and Costal Zones, South China Botanical Garden, Chinese Academy of Sciences, Guangzhou 510650, China; wxj@scbg.ac.cn; 5 Xiaoliang Research Station for Tropical Coastal Ecosystems, South China Botanical Garden, Chinese Academy of Sciences, Guangzhou 510650, China; 6 Center of Economic Botany, Core Botanical Gardens, Chinese Academy of Sciences, Guangzhou 510275, China

**Keywords:** *Millettia pinnata*, calmodulin-like, salt tolerance, heterologous expression

## Abstract

Salt stress is a major increasing threat to global agriculture. Pongamia (*Millettia pinnata*), a semi-mangrove, is a good model to study the molecular mechanism of plant adaptation to the saline environment. Calcium signaling pathways play critical roles in the model plants such as Arabidopsis in responding to salt stress, but little is known about their function in Pongamia. Here, we have isolated and characterized a salt-responsive *MpCML40*, a calmodulin-like (CML) gene from Pongamia. MpCML40 protein has 140 amino acids and is homologous with Arabidopsis AtCML40. MpCML40 contains four EF-hand motifs and a bipartite NLS (Nuclear Localization Signal) and localizes both at the plasma membrane and in the nucleus. *MpCML40* was highly induced after salt treatment, especially in Pongamia roots. Heterologous expression of *MpCML40* in yeast cells improved their salt tolerance. The *35S::MpCML40* transgenic Arabidopsis highly enhanced seed germination rate and root length under salt and osmotic stresses. The transgenic plants had a higher level of proline and a lower level of MDA (malondialdehyde) under normal and stress conditions, which suggested that heterologous expression of *MpCML40* contributed to proline accumulation to improve salt tolerance and protect plants from the ROS (reactive oxygen species) destructive effects. Furthermore, we did not observe any measurable discrepancies in the development and growth between the transgenic plants and wild-type plants under normal growth conditions. Our results suggest that MpCML40 is an important positive regulator in response to salt stress and of potential application in producing salt-tolerant crops.

## 1. Introduction

Salt stress is one of the significant environmental factors affecting plant growth and productivity. Soil salinization is a fast-growing global problem, especially in the arid and semi-arid areas of the world [1]. Moreover, lots of arable lands are changing to salinized land now due to the rising sea level, so improving salt tolerance of crops by genetic modification is a critical aspect of crop breeding. Halophytes are kinds of plants growing in high salinity (salt concentration is around 200 mM NaCl or more) conditions where most crops cannot survive [2]. Mangroves are trees or large shrubs which grow within the intertidal zone in tropical and subtropical regions. Mangrove species include true mangroves and semi-mangroves (also called mangrove associates). True mangroves have morphological specialization, such as aerial roots and vivipary; physiological mechanism for salt exclusion and/or salt excretion [3]. Pongamia (*Millettia pinnata* syn. *Pongamia pinnata*) is a semi-mangrove plant that can grow in either freshwater or moderate salinity water [3]. Unlike true mangroves, Pongamia does not have the salty glands or other specialized morphological traits to endure salinity stress, which suggested that its salt tolerance may be more attributed to gene regulation and protein function [3]. Therefore, investigating the molecular mechanisms of Pongamia salt tolerance may provide promising strategies for crop breeding by genetic modification.

Pongamia is a diploid legume (2n = 22) with a genome size of ~1300 Mb, which is ten times that of Arabidopsis [4,5]. Several transcriptome analyses of Pongamia root, leaf, flower, pod, and seedling have been reported in recent years [6,7,8,9,10,11]. However, only a few Pongamia functional genes have been identified and characterized. Four circadian clock genes (*ELF4, LCL1, PRR7,* and *TOC1*) were identified from Pongamia using soybean as the reference [12]. A stearoyl-ACP desaturase (SAD) was isolated from Pongamia seeds and suggested a seed development function [13]. Furthermore, two other desaturase genes, *MpFAD2-1* and *MpFAD2-2* (Fatty Acid Desaturase 2), were also isolated and characterized [14]. Although 23,815 candidate salt-responsive genes were identified from Pongamia by comparing the expression pattern under seawater and freshwater treatments using Illumina sequencing, so far, only one report showed that a chalcone isomerase gene, *MpCHI,* enhanced the salt tolerance of yeast (*Saccharomyces cerevisiae*) salt-sensitive mutants [7,15].

The calcium ion Ca^2+^ is a second messenger in all eukaryotes. It is perceived by several Ca^2+^ binding proteins, including calmodulin (CaM), calmodulin-like protein (CML), calcineurin B-like (CBL), and calcium-dependent protein kinase (CPK or CDPK) [16,17,18]. CaMs are present in all eukaryotes, but CMLs, CBLs, and CPKs are only identified in plants and some protists [17,19]. The most common Ca^2+^ binding motif is the EF-hand motif present in most Ca^2+^ binding proteins, including CaMs and CMLs [20,21]. In Arabidopsis, there are only six typical *CaM* genes, while over 50 *CML* genes have been identified [22]. All six AtCaMs have very similar protein sequences with 149 amino acids containing four EF-hand motifs. In contrast, AtCMLs have 80–330 amino acids with two to four EF-hand motifs [22]. Many *CML* genes have been reported to be involved in abiotic stress signaling [23,24]. In Arabidopsis, it was reported that the expression levels of *AtCML12* (also called *TCH3*) and *AtCML24* (also called *TCH2*) were highly enhanced under heat stress [24,25]. The CML::GUS report gene data showed that *AtCML37, AtCML38*, and *AtCML39* genes were induced by several stimuli, including salt and drought stress [26]. AtCML15 (also called CaM15) could interact with Na^+^/H^+^ exchanger 1 (AtNHX1) and played roles in maintaining cellular pH and ion homeostasis [27]. *AtCML9* and *AtCML24* genes were induced by abiotic stress and abscisic acid (ABA) and functioned in response to ABA and salt or ion stress [28,29]. *AtCML20* could negatively modulate ABA signaling and drought response [30]. *AtCML42* was reported to function in both herbivory defense and ABA-mediated drought stress response [31]. A rice CML gene, *OsMSR2*, could increase drought or salt tolerance and ABA sensitivity in Arabidopsis [32]. In addition to Arabidopsis and rice, several studies showed that CMLs in other plants were also involved in abiotic stress signaling. Expression of 32 CML genes in wild-growing grapevine (*Vitis amurensis*) was shown to be responsive to abiotic stresses, including drought, salt, heat, and cold [33]. In *Glycine soja*, *GsCML27* participated in salt and osmotic stresses [34]. In *Medicago truncatula, MtCML40* was involved in salt stress [35]. In *Camellia sinensis*, *CsCML16*, *CsCML18-2*, and *CsCML42* were induced by cold and salt conditions, while *CsCML38* was induced by drought and ABA treatments [36].

In the present study, we identified a *CML* gene, *MpCML40*, from Pongamia. The MpCML40 protein contained a typical EF-hand motif. Under salt treatment, the *MpCML40* gene was highly induced in roots. Heterologous expression of *MpCML40* in Arabidopsis strongly enhanced the salt tolerance of transgenic plants.

## 2. Results

### 2.1. MpCML40 Is an EF-Hand Motif-Containing Calmodulin-Like Protein

**Figure 1:**
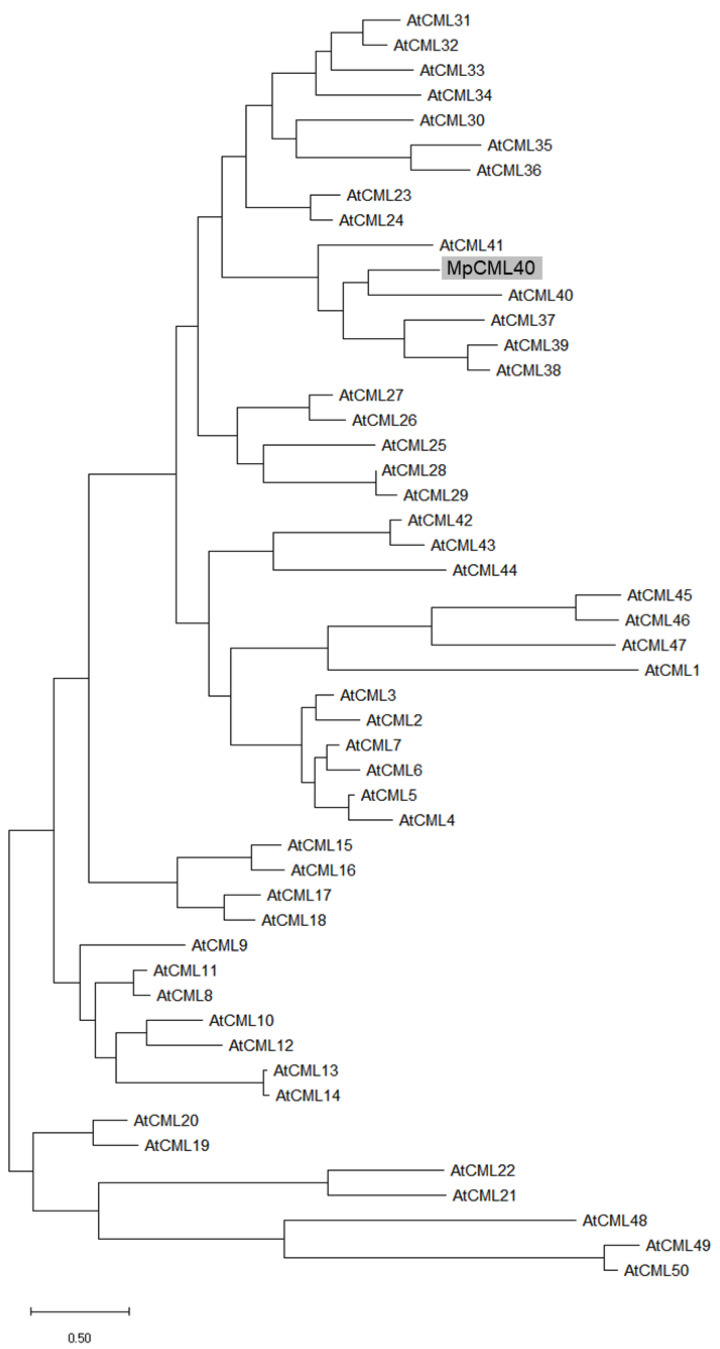
Phylogenetic tree of MpCML40 with Arabidopsis CML proteins. MpCML40 protein and 50 Arabidopsis calmodulin-like (CML) proteins were used for phylogenetic analysis [22]. MpCML40 was marked by a grey box.

**Figure 2:**
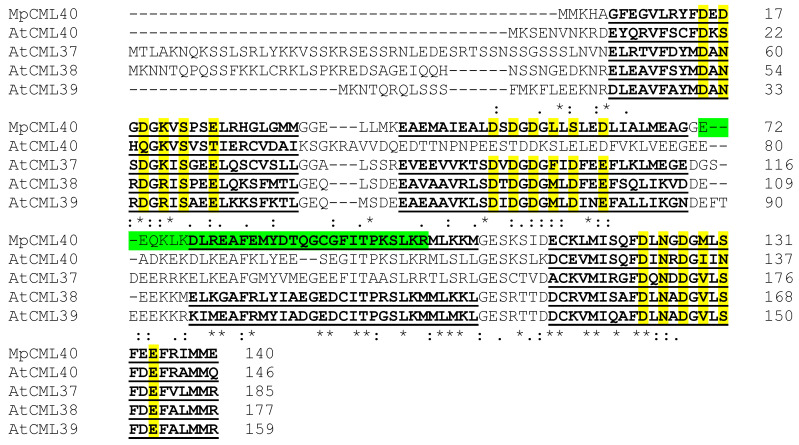
Amino acid sequence alignment of MpCML40, AtCML37, AtCML38, AtCML39, and AtCML40. The EF-hand motifs were underlined, and the highly conserved amino acids in EF-hand motifs were highlight by yellow color. A predicted bipartite Nuclear Localization Signal (NLS) was highlighted by green color.

The full-length cDNA of the *MpCML* gene was obtained by 5′ and 3′ rapid amplification of cDNA ends (RACE) assay with four specific internal primers based on the sequence of an EST from our previous study [7]. The full-length sequence of cDNA, which was deposited in GenBank under accession number MW650864, comprised a 423 bp open reading frame (ORF), 135 bp 5′ untranslated region (UTR), and 258 bp 3′ UTR followed by a polyA tail (Table 1). The corresponding protein contained 140 amino acids. A phylogenetic tree based on the amino acid sequences of MpCML and 50 Arabidopsis CML proteins revealed that this MpCML protein was homologous with AtCML40 (Figure 1). Therefore, we named it MpCML40. In addition to AtCML40, the sequence of MpCML40 was also similar to some other members in this subfamily, such as AtCML37, AtCML38, and AtCML39 (Figure 1). Based on SMART (Simple Modular Architecture Research Tool) analysis, MpCML40 also had four EF-hand motifs (Figure 2), the same as AtCML38 and AtCML39. However, the third EF-hand motif lacked the conserved 12 residues with the pattern X•Y•Z•–Y•–X••–Z (also called DxDxDG loop), which might be a pseudo EF-hand motif as previously reported [21,37,38]. Moreover, a predicted bipartite NLS (Nuclear Localization Signal) was found in the third EF-hand motif (Figure 2), which indicated MpCML40 might at least partially localize in the nucleus.

### 2.2. MpCML40 Localizes at the Plasma Membrane and in the Nucleus

**Figure 3:**
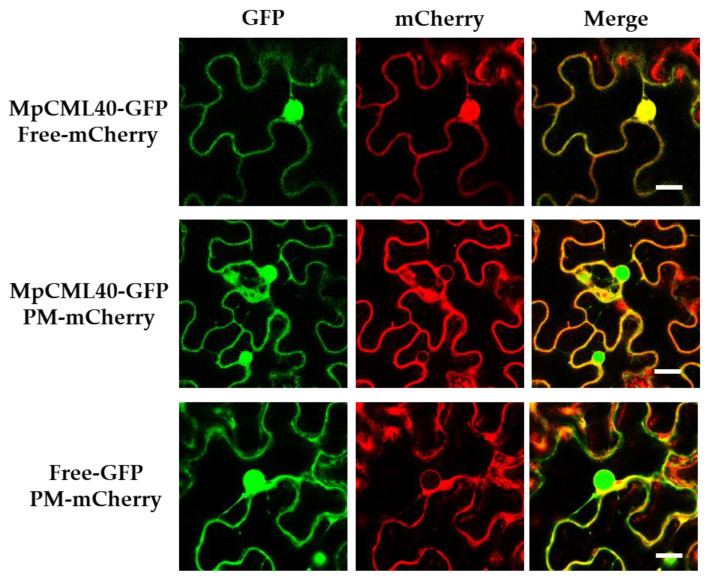
Subcellular localization of MpCML40-GFP. Subcellular localization of MpCML40-GFP was assayed with plasma membrane (PM-mcherry) marker or free mcherry in tobacco leaf epidermal cells. The fluorescence signals were detected 48 h after infiltration. Bar = 20 μM. The experiments were repeated two times with similar results.

MpCML40 was predicted to have a bipartite NLS by the cNLS Mapper online tool with a score of 6.7, which indicated that this protein might partially localize in the nucleus [39,40,41]. To verify the subcellular localization of this protein, MpCML40-GFP was expressed in tobacco (*Nicotiana benthamiana*) leaves by *Agrobacterium tumefaciens*-mediated transient expression system. The results showed that MpCML40 was indeed partially localized in the nucleus in tobacco epidermal cells, colocalized with free mCherry (Figure 3). Besides, MpCML40-GFP was also colocalized with the specific plasma membrane (PM) mCherry marker, with free GFP as a negative control (Figure 3). These results illustrated MpCML40 localized both at the plasma membrane and in the nucleus in tobacco epidermal cells.

### 2.3. MpCML40 Gene Is Highly Induced by Salt Stress in Pongamia Roots

**Figure 4:**
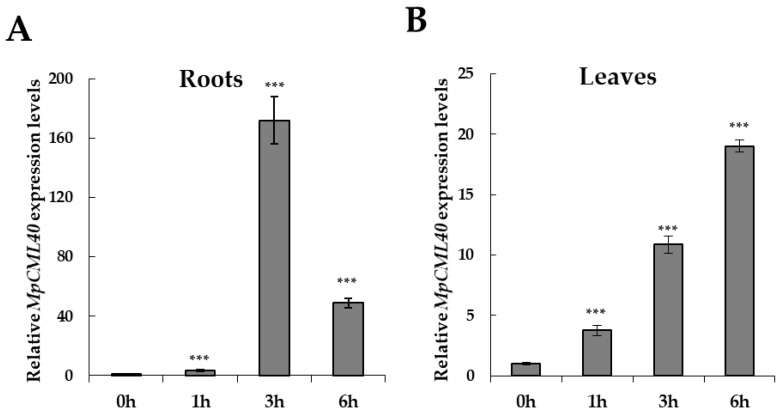
Gene expression changes of *MpCML40* in Pongamia roots and leaves upon salt stress. Relative expression levels of the *MpCML40* gene in roots (**A**) and leaves (**B**) after 500 mM NaCl treatment were analyzed by qRT-PCR using primers listed in Table 2. *MpActin* gene was used as an internal reference. Error bars show mean values (±SD) of three independent samples. *** *p* < 0.001 (Student’s *t*-test).

The *AtCML37*, *AtCML38*, and *AtCML39* genes were all expressed in Arabidopsis root and highly accumulated after salt treatments [26]. To analyze the spatial and temporal expression pattern of *MpCML40* under salt stress, one-month-old Pongamia seedlings were subjected to qRT-PCR analyses. The relative expression level of *MpCML40* was significantly increased in roots and leaves after salt treatments (Figure 4). Especially in the root, *MpCML40* was strongly up-regulated at three and six hours after salt treatment (Figure 4). These results demonstrated that *MpCML40* was a salt-responsive gene.

### 2.4. The pYES22-MpCML40-Transformed Yeast Has Enhanced Salt Tolerance

**Figure 5:**
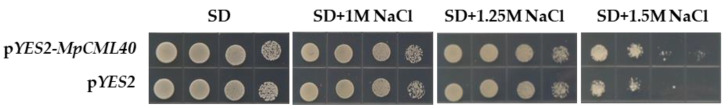
Salt tolerance of the *pYES2-MpCML40* transformed yeast. Series dilutions (1, 10, 100, 1000 folds) of the *pYES2-MpCML40* transformed yeast and *pYES2* (empty control) transformed yeast were grown on SD/-Ura agar plate containing different concentrations of NaCl (1 M, 1.25 M, 1.5 M).

We used yeast to preliminarily study the function of *MpCML40* in salt stress response. The *S. cerevisiae* strain W303 was transformed with either *pYES2-MpCML40* plasmid or *pYES2* empty vector. The positive colonies were transferred to the SD/-Ura agar plates with different concentrations of NaCl. The yeast strains showed resistance to low concentrations of NaCl. However, when the concentration increased to 1.5 M, the yeasts with *pYES2-MpCML40* plasmid grew faster than those with empty vectors (Figure 5), which suggested that *MpCML40* could substantially improve the salt tolerance of yeast.

### 2.5. Heterologous Expression of MpCML40 in Arabidopsis Strongly Enhances Salt and Osmotic Tolerance

**Figure 6:**
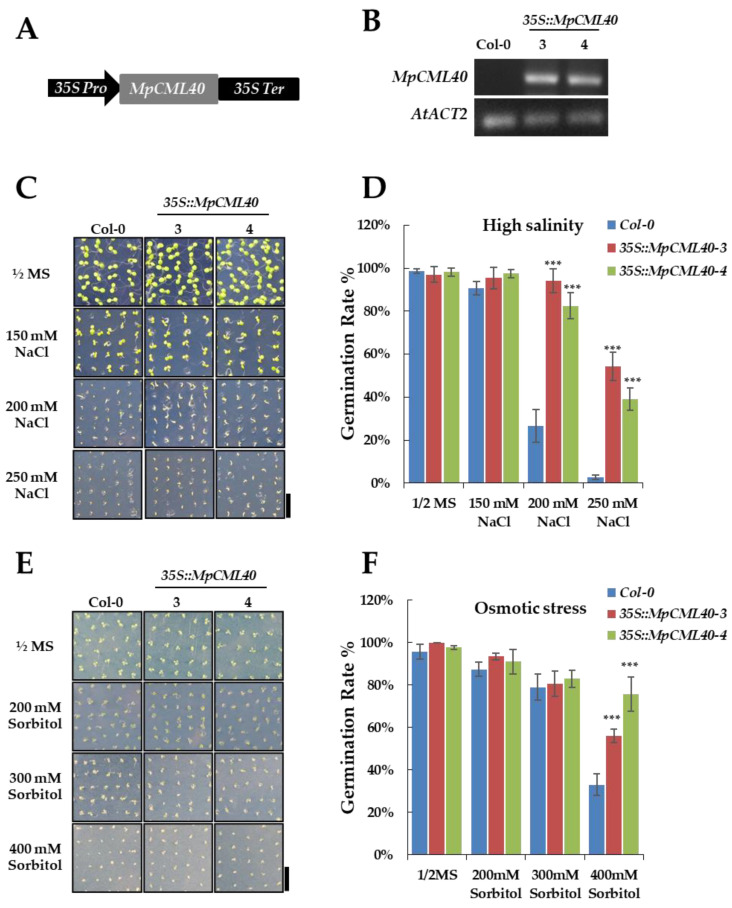
Effects of *MpCML40* on seed germination rate under salt and osmotic stress. (**A**) Schematic structure of the *MpCML40* expression construct. *35S Pro*, cauliflower mosaic virus 35S promoter. *35S Ter*, cauliflower mosaic virus 35S terminator. (**B**) Expression levels of the *MpCML40* gene in the wild-type and transgenic Arabidopsis plants were analyzed by RT-PCRs using the *MpCML40* full-length primers listed in Table 2. *AtACT2* (*ACTIN2*) was used as a control. (**C**–**F**) Typical phenotype and germination rate of wild-type and transgenic Arabidopsis seeds germinated on ½ MS medium containing different concentrations of NaCl (**C**,**D**) and sorbitol (**E**,**F**). Bar = 1 cm. Error bars show mean values (±SD) of germination rate of three independent plates. *** *p* < 0.001 (Student’s *t*-test).

To investigate the possible functions of *MpCML40* in salt stress response, we generated the transgenic Arabidopsis plants carrying *35S-Pro::MpCML40* (Figure 6A). Two independent transgenic lines were identified and validated by RT-PCR (Figure 6B). The growth and development phenotype of the transgenic plants were very similar to wild-type plants. We first checked the seeds; germination rate for the *35S::MpCML40* transgenic and wild-type Arabidopsis under salt stress. High concentrations (200 mM and 250 mM) of NaCl strongly inhibited seed germination of wild-type plants, whereas 200 mM of NaCl had no significant effects on the transgenic plants (Figure 6C,D). Moreover, the seeds of the transgenic plants showed obviously higher germination rate on the medium containing 250 mM of NaCl compared with wild-type plants (Figure 6C,D). We also assayed the seed germination rate under osmotic stress. Under low concentrations (200 mM and 300 mM) of sorbitol, the seeds of both wild-type and transgenic plants had high germination rates (Figure 6E,F). However, the seed germination rate was significantly higher in transgenic plants than in wild-type plants under high concentration (400 mM) of sorbitol (Figure 6E,F).

**Figure 7:**
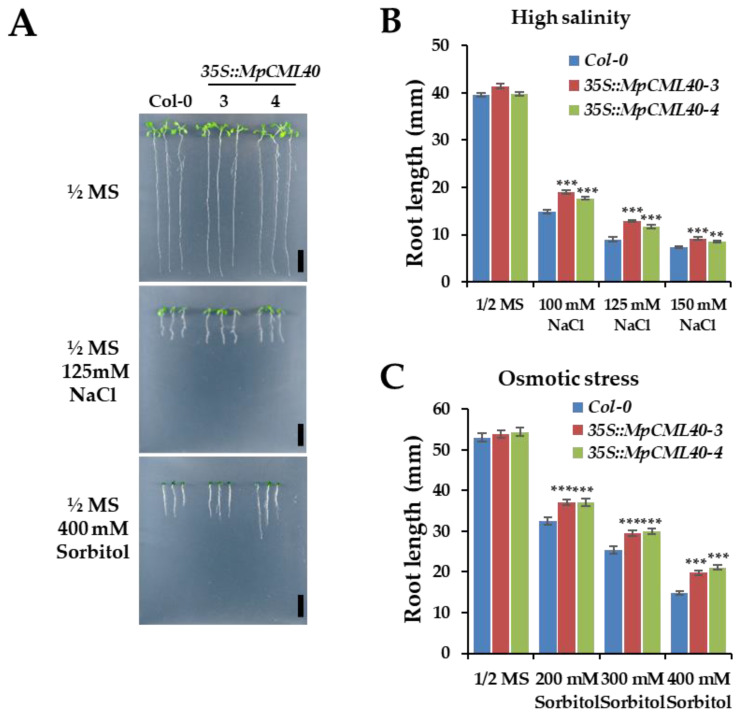
Effects of *MpCML40* on root growth under salt and osmotic stress. (**A**) Typical root length phenotype of two-week wild-type and *MpCML40* heterologously expressing Arabidopsis seedlings grown on ½ MS medium containing 125 mM of NaCl and 400 mM of sorbitol. (**B**,**C**) The root length of two-week wild-type and transgenic Arabidopsis seedlings grown on ½ MS medium containing different NaCl and sorbitol concentrations. Bar = 1 cm. Error bars show mean values (±SE) of germination rate from 50 independent seedlings. ** *p* < 0.01, *** *p* < 0.001 (Student’s *t*-test).

Secondly, the root lengths of wild-type and transgenic seedlings were measured under salt and osmotic stress. The seedlings were germinated and grown for three days on a normal ½ MS agar medium and then transferred to the medium containing different NaCl or sorbitol concentrations. After ten days of treatments, the root lengths of both wild-type and transgenic seedlings were inhibited (Figure 7A–C). However, the roots of the transgenic seedlings were significantly longer than those of wild-type seedlings under salt and osmotic stress (Figure 7B,C).

**Figure 8:**
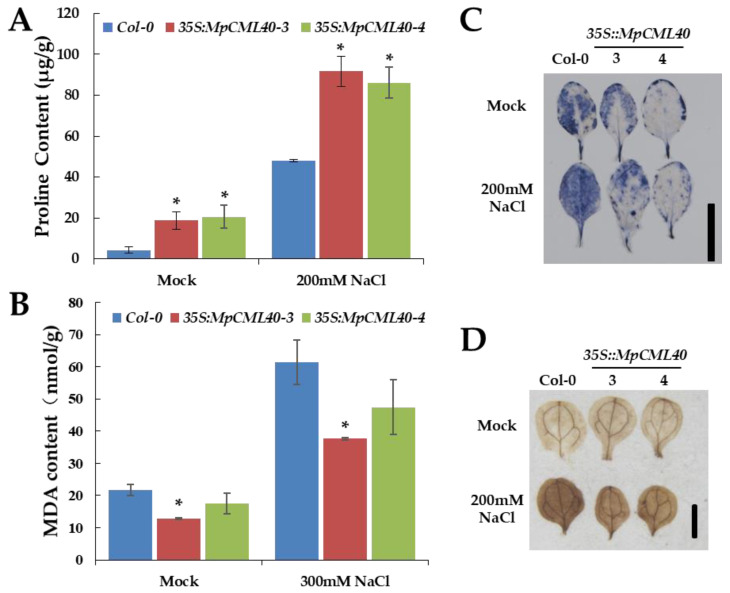
Effects of *MpCML40* heterologous expression on salt-stress-related metabolites. (**A**) Proline content of wild-type and *35S::MpCML40* Arabidopsis plants. (**B**) Micro Malondialdehyde (MDA) content of wild-type and *35S::MpCML40* plants. Error bars show mean values (±SD) of germination rate from three independent samples. * *p* < 0.05 (Student’s *t*-test). (**C**) NBT staining of wild-type and *35S::MpCML40* four-week-old Arabidopsis rosette leaves. Bar = 1 cm. (**D**) DAB staining of wild-type and *35S::MpCML40* two-week-old Arabidopsis cotyledons grown on ½ MS agar plates. Bar = 1 mm.

Lastly, we measured the levels of two critical stress-associated metabolites, malondialdehyde (MDA) and proline, under mock or 200 mM NaCl conditions. Compared with wild-type plants, the transgenic ones showed a significantly higher level of proline and a slightly lower level of MDA (Figure 8A,B). To analyze the reactive oxygen species (ROS) induced by salt stress, nitroblue tetrazolium (NBT) and 3,3’-diaminobenzidine (DAB) staining were performed to detect the contents of H_2_O_2_ and O_2_^−^ under mock or 200 mM NaCl condition. The leaves of transgenic plants showed lighter staining color than those of wild-type plants did (Figure 8C,D), indicating lower contents of H_2_O_2_ and O_2_^−^ in transgenic plants under salt stress.

## 3. Discussion

As a semi-mangrove, Pongamia usually grows under high salinity conditions, which confer this species a unique genetic resource for exploring salt-responsive genes and investigating molecular mechanisms of plant salt tolerance. From 23,815 candidate salt-responsive genes formerly identified in Pongamia using Illumina sequencing [7], we identified a salt-induced CML gene, *MpCML40*, and characterized its function in the process of the salt stress response by heterologous expressing this gene in yeast and Arabidopsis. Our results showed that heterologous expression of *MpCML40* in yeast could improve its salt tolerance. The *35S::MpCML40* transgenic Arabidopsis did not show any growth and development phenotype compared with wild-type plants under normal growth conditions but had a higher germination rate and root length under salt stress. Our findings supported that *MpCML40* played a critical role in salt stress response.

### 3.1. MpCML40 Is a Salt-Induced CML Gene

CMLs are novel plant-specific Ca^2+^ sensors. There were 50 and 32 CML genes in Arabidopsis and rice, respectively [22,42]. CML genes were recently also identified in tomato, Medicago, grapevine, and apple [33,43,44,45,46,47]. *MpCML40* is the first CML gene identified in Pongamia. The phylogenetic tree showed that *MpCML40* was homologous with AtCML40 (Figure 1). Meanwhile, *MpCML40* was predicted to contain four EF-hand motifs, which were similar to AtCML38 and AtCML39 (Figure 2). Judging from the bipartite NLS, *MpCML40* might be present in the nucleus and also at the plasma membrane (Figure 2 and Figure 3). CML genes in Arabidopsis, rice, and other plants were reported to be induced by several abiotic stresses. In our study, the expression level of *MpCML40* was highly enhanced upon salt stress, especially in roots at three and six hours after treatment (Figure 4). These results were consistent with the previous report that the leaves and roots of Pongamia had differential responses to salt stress while the roots were more efficient than the leaves [48].

### 3.2. MpCML40 Improves Salt Tolerance in Both Yeast and Arabidopsis

A NaCl-induced gene from Pongamia, *MpCHI*, was formerly reported to enhance salt tolerance of yeast [15]. Accordingly, we transformed *MpCML40* into yeast cells and assessed their salt responses. The wild-type yeast strain could grow in a high salt concentration medium, but the *MpCML40*-transformed yeasts grew obviously better than wild-type yeasts under 1.5 M of NaCl (Figure 5). Furthermore, we heterologously expressed *MpCML40* in Arabidopsis and conducted phenotype analysis. The *35S::MpCML40* transgenic plants had nearly normal germination rate under 200 mM NaCl condition. In contrast, the wild-type plants had significantly lower germination rates (Figure 6C,D). As NaCl concentration increased to 250 mM, nearly no seeds of wild-type plants germinated, while the seeds of transgenic plants still had a germination rate of about 50% (Figure 6C,D). In addition, the transgenic plants also showed a better germination rate than wild-type plants did under high concentrations of sorbitol (Figure 6E,F). Under both salt and osmotic stresses, the transgenic seedlings exhibited longer roots (Figure 7).

Halophytes are considered as one of the best germplasms for identifying salt-responsive genes, but only a few genes that could improve salt tolerance were isolated and characterized from mangroves, especially semi-mangroves [49]. Here, we found *MpCML40* could improve the salt tolerance of transgenic Arabidopsis. Moreover, to evaluate a genetic modification plant, it is essential to check whether or not the growth and development of the transgenic plant have been affected without stress conditions [2]. The *35S::MpCML40* transgenic plants did not show any visible growth and development retardation under normal conditions.

Proline is an important osmolyte for stabilizing macromolecules and membranes in the cell, and a higher level of proline can protect plant cells under salt and osmotic stresses [50,51,52]. Our results showed that the *35S::MpCML40* transgenic Arabidopsis had a higher level of proline even before salt treatment (Figure 8A), which revealed that MpCML40 could contribute to proline accumulation for salt tolerance. MDA is the main product of membrane lipid peroxidation under salt stress. Hence, the MDA level was regarded as an indicator of cell membrane damage [52]. The lower levels of MDA in the *MpCML40*-heterologous expressing plants supported the potential roles of *MpCML40* in salt stress response (Figure 8B). Besides, the light NBT and DAB staining color in transgenic plants revealed reduced contents of ROS under salt stress (Figure 8C,D). Taken together, our results uncovered that heterologous expression of *MpCML40* in Arabidopsis might contribute to proline accumulation to enhance salt tolerance of the transgenic plants and protect them from the ROS destructive effects.

## 4. Materials and Methods

### 4.1. Plant Materials and Growth Conditions

*Arabidopsis thaliana* ecotype Columbia (Col-0) was used as the genetic background of the *35S::MpCML40* transgenic plants. The transgenic plants were generated by agrobacterium-mediated floral dipping method and selected by BASTA (glufosinate ammonium) resistance. The T3 generation of homozygous plants were used for phenotype analysis. The plants were grown on soils and cultivated in the reach-in growth chamber with 16 h light at 22 °C and 8 h dark at 22 °C with approximately 120 μmol·m^−2^·s^−1^ of fluorescent white light. Arabidopsis seedlings were germinated and cultivated on ½ MS agar plates (½ × MS basal salts including 1% sucrose and 0.8% agar) with 16 h light at 22 °C and 8 h dark at 22 °C. Pongamia seeds were soaked in tap water at 28 °C in a growth cabinet until radicle appeared. These germinated seeds were then planted in soil for further growth.

### 4.2. Full-Length cDNA Cloning, Phylogenetic Analysis, and Motif Prediction

The highly conserved region of the unigene from the Pongamia transcriptome was used as a template for designing gene-specific internal primers for 5′ and 3′ RACE assay using SMARTer^TM^ RACE cDNA Amplification Kit (Takara Bio, Madison, WI, USA). The total RNA was isolated from one-month-old Pongamia leaves. All primers used were listed in Table 2. The 5′ and 3′ ends of cDNA were sequenced and assembled into full-length cDNA. MpCML40 protein and 50 Arabidopsis CML proteins were used for phylogenetic analysis [22]. The phylogenetic tree was constructed using the Maximum Likelihood method implemented in the MEGA X program [53,54]. Alignment of MpCML40, AtCML37, AtCML38, AtCML39 and AtCML40 was conducted with Clustal Omega [55]. The conserved EF-hand motifs were analyzed by SMART (Simple Modular Architecture Research Tool) [56,57]. The NLS was searched by cNLS Mapper [39,40].

### 4.3. Subcellular Localization

PIP2A (plasma membrane intrinsic protein 2A) was used as the mCherry tagged plasma membrane marker (PM-mCherry). The agrobacterial strain GV3101 containing PM marker-mCherry or free mCherry constructs was used at OD600 = 0.5, and GV3101 containing MpCML40-GFP or free-GFP constructs was used at OD600 = 0.25. The four-week-old *N. benthamiana* leaves were used for agrobacteria-mediated transient expression. The images were taken at 48 h after infiltration using LEICA SP8 STED 3X fluorescence microscope confocal system.

### 4.4. Quantitative Real-Time PCR

One-month-old Pongamia seedlings were transferred from soil into ½ MS liquid medium. After overnight culture, the normal ½ MS liquid medium was replaced by ½ MS liquid medium containing 500 mM NaCl. The seedling samples were collected at 0, 1, 3, 6 h after treatments for total RNA extraction using TRIzol™ Plus (Takara Bio, Madison, Wisconsin, USA) following the manufacturer’s protocol. About 1000 ng of total RNA was digested by DNase I for 30 min at 37 °C before reverse transcription. DNase digestion was terminated by addition of 25 mM EDTA and followed by incubation at 70 °C for 10 min. First strand cDNA synthesis was performed using an oligo(dT) 18 primer and the GoScript™ Reverse Transcriptase (Promega, Madison, Wisconsin, USA). Subsequently, qRT-PCR was performed on Roche LightCyler 480 with gene-specific primers and SYBR Green (Life Technologies, Rockville, Maryland, USA). All primers used in qRT-PCR were listed in Table 2.

### 4.5. Yeast Transformation and Growth Assay

The yeast strain W303 was used for growth assay. The yeast cells were cultured at 30 °C with shaking at 230 rpm and collected at the OD600 0.4–0.6. The yeast cells were centrifuged at 1000 rpm for 5 min at room temperature. The supernatants were discarded, and the cell pellets were suspended in sterile water. The cells were pooled into one tube (final volume 25–50 mL) and centrifuged at 1000 rpm for 5 min at room temperature. The cell pellets were suspended in 1.5 mL freshly prepared and sterile 1× TE/1× LiAc solutions (10 mM Tris-HCl; 1 mM EDTA; pH 7.5; 100 mM LiAc). Next, 0.5 µg plasmid DNA and 0.1 mg salmon sperm carrier DNA were added into 100 µL competent cells in a fresh 1.5 mL tube and mixed by vortexing. The 600 µL freshly prepared and sterile PEG/LiAc solutions (40% PEG 4000; 10 mM Tris-HCl; 1 mM EDTA; pH 7.5; 100 mM LiAc) were added to each tube and vortexed at high speed for 10 s. The mixture was incubated at 30 °C for 30 min with shaking at 200 rpm. Then, 70 µL DMSO was added and mixed well by gentle inversion (Do not vortex). The mixture was heat-shocked for 15 min in a 42 °C water bath and then transferred on ice for 1–2 min. The cells were centrifuged for 30 s at 6000 rpm at room temperature. The supernatants were removed, and the cell pellets were re-suspended in 0.5 mL sterile 1× TE buffer (10 mM Tris-HCl; 1 mM EDTA; pH 7.5). Later, 100–500 µL of suspended cells were spread on each SD/-Ura selection agar plate and incubated at 30 °C until colonies appeared. The positive colonies were cultured overnight and then diluted to 1, 10, 100, 1000 folds. 5 µL of diluted yeast cells were transferred to SD/-Ura agar plate containing different concentrations of NaCl and incubated at 30 °C until colonies appeared.

### 4.6. Phenotype Analysis of Wild-Type and 35S::MpCML40 Transgenic Arabidopsis Plants

For germination rate assays, at least 100 seeds of wild-type and transgenic plants were sowed on ½ MS medium containing different concentrations of NaCl (150, 200, and 250 mM) and sorbitol (200, 300, and 400 mM). After three days of vernalization at 4 °C in the dark, the seeds were transferred to light for the assessments of germination rates. A seed was considered as germinated when the radical protruded through its envelope.

For root length assay, the seedlings were germinated and grown for three days on normal ½ MS agar medium and then transferred to the medium containing different concentrations of NaCl (100, 125, 150 mM) and sorbitol (200, 300, 400 mM). The root length of at least 50 seedlings was measured by ImageJ software after 10 days of treatments.

### 4.7. Proline Content Measurement

Proline content was measured using Proline (PRO) Content Assay Kit (BC0290, Solarbio, Beijing, China) following the manufacturer’s protocol. Briefly, 10-day-old seedlings from ½ MS agar plates were transferred into ½ MS liquid medium at 12 h before treatments and then treated overnight with 200 mM of NaCl. 100 mg of seedlings were weighted and homogenized with 1 mL of extraction buffer in mortar on ice. Then, the extraction procedure was followed the manufacturer’s protocol. Proline content was determined by reading the optical density of the sample at 520 nm using a spectrometer.

### 4.8. MDA Content Measurement

MDA content was measured using Micro Malondialdehyde (MDA) Assay Kit (BC0020, Solarbio, Beijing, China) following the manufacturer’s protocol. Briefly, four-week-old plants in soil were treated with 300 mM of NaCl for one week. 100 mg of seedlings were weighted and homogenized with 1 mL of extraction buffer in mortar on ice. Then, the extraction procedure was followed the manufacturer’s protocol. MDA content was determined by reading the optical density of the sample at 600 nm, 532 nm, 450 nm using a spectrometer.

### 4.9. DAB and NBT Staining

Two-week-old Arabidopsis cotyledons grown on ½ MS agar plate were used for DAB staining, and four-week-old rosette leaves were used for NBT staining. The leaves were vacuumed in ½ MS liquid medium containing 200 mM of NaCl for 5 min and then soaked for another 4 h. Staining was performed by vacuuming the leaves in 1 mg/mL DAB solution or 0.2% NBT solution for 5 min and then staining for another 4 h.

## 5. Conclusions

In conclusion, we isolated and functionally characterized a gene encoding a calmodulin-like protein, *MpCML40*, from Pongamia. The *35S::MpCML40* transgenic Arabidopsis accumulated high levels of prolines and was more tolerant to salt and osmotic stress than wild-type Arabidopsis, suggesting that MpCML40 was a positive regulator in response to salt stress. Importantly, the transgenic plants grew and developed as well as wild-type plants under normal conditions. Our findings improved the understandings of salt-responsive mechanisms in Pongamia and also provided a potential candidate for crop breeding by genetic modification.

## Figures and Tables

**Table 1:** The nucleotide sequence of *MpCML40* cDNA. The open reading frame (ORF) of *MpCML40* is highlighted in yellow.

1	CCACAACATA TAATAACAAC TCAATTTTCC ATTTGCATAC AAGTTACATT TTCTCCTTCT
61	TATTCTTCTT GTTATTGTGT ACATTAAAGA TTTGAACAAA TTACACTACA CCTTTAAGAT
121	AAGGAGCATT GTAACATGATGAAGCATGCG GGTTTTGAGG GTGTTCTTCG ATATTTTGAT
181	GAAGATGGGG ATGGAAAGGT TTCACCTTCA GAGTTAAGGC ATGGATTGGG AATGATGGGT
241	GGGGAGCTTT TGATGAAAGA AGCAGAGATG GCAATTGAGG CACTGGATTC TGATGGTGAT
301	GGGTTGTTGA GTTTGGAGGA TTTGATTGCT TTGATGGAAG CAGGGGGAGA GGAACAAAAG
361	TTGAAGGATT TGAGAGAAGC TTTTGAGATG TATGACACTC AAGGGTGCGG ATTTATAACC
421	CCAAAGAGCT TGAAGAGGAT GCTTAAGAAG ATGGGAGAGT CCAAGTCCAT TGATGAATGC
481	AAATTGATGA TTAGTCAATT TGATTTGAAT GGGGATGGGA TGCTTAGCTT TGAAGAATTC
541	AGAATTATGA TGGAGTGAGG CCAGTATATT TGTTGATGAT ATTGTTTAGT TTGTTTGTTT
601	GTTTGGGAGG AAGAGGGGTA TAGTTAAGTG GATTTGATTT ATTTGTTTGC AGTTTGCACA
661	TGTATAAATA ACTCCTTTTG TGCTTTGCAA TACTTTTGAC AATTGATTAA CTGTTAGATT
721	TCTCCCAAGT TCCTACATAA AAAATTATTC AAATTTTCTT AATGGGAGTT GTATTATGAC
781	TATTATCATG GTTAAATATA TTTTTTATTT CGTTCCAAAA AAAAAAAAAA AAAAAAAAAA

**Table 2:** The list of primer sequences.

Primer	Primer Sequence (5′ → 3′)
MpActin.RtF	AGAGCAGTTCTTCAGTTGAG
MpActin.RtR	TCCTCCAATCCAGACACTAT
MpCML40.RtF	GCACTGGATTCTGATGGTGATGGG
MpCML40.RtR	GCTCTTTGGGGTTATAAATCCGCA
MpCML40.3’GSP	TGGCAATTGAGGCACTGGATTCTGATGG
MpCML40.3’NGSP	ATGGAAGCAGGGGGAGAGGAACAA
MpCML40.5’GSP	TCCGCACCCTTGAGTGTCATACATCTCA
MpCML40.5’NGSP	CCTCCAAACTCAACAACCCATCACCATC
MpCML40.yeastF	CGCGGATCCATGATGAAGCATGCGGGTTTTGAGG
MpCML40.yeastR	CCGCTCGAGTCACTCCATCATAATTCTGAA
MpCML40.plantF	TATGGCGCGCCCATGATGAAGCATGCGGGTTTTGA
MpCML40.plantR	CTCCATCATAATTCTGAATTCTT
AtACT2.RtF	GACCTTTAACTCTCCCGCTATG
AtACT2.RtR	GAGACACACCATCACCAGAAT

## Data Availability

MpCML40 sequence data is available in GenBank.
